# Effect of Sodium Valproate on the Conformational Stability of the Visual G Protein-Coupled Receptor Rhodopsin

**DOI:** 10.3390/molecules26103032

**Published:** 2021-05-19

**Authors:** Neda Razzaghi, Pol Fernandez-Gonzalez, Aina Mas-Sanchez, Guillem Vila-Julià, Juan Jesus Perez, Pere Garriga

**Affiliations:** 1 Grup de Biotecnologia Molecular i Industrial, Centre de Biotecnologia Molecular, Departament d’Enginyeria Química, Universitat Politècnica de Catalunya-Barcelona Tech, Edifici Gaia, Rambla de Sant Nebridi 22, 08222 Terrassa, Spain; neda.razzaghi@upc.edu (N.R.); pol.fernandez.gonzalez@upc.edu (P.F.-G.); aina.mas.sanchez@upc.edu (A.M.-S.); 2 Grup de Biotecnologia Molecular i Industrial, Centre de Biotecnologia Molecular, Departament d’Enginyeria Química, Universitat Politècnica de Catalunya-Barcelona Tech., Avinguda Diagonal, 647, 08028 Barcelona, Spain; guillem.vila.julia@upc.edu (G.V.-J.); juan.jesus.perez@upc.edu (J.J.P.)

**Keywords:** rhodopsin, G protein-coupled receptor, sodium valproate, retinitis pigmentosa, conformational stability

## Abstract

Rhodopsin is the G protein-coupled receptor of rod photoreceptor cells that mediates vertebrate vision at low light intensities. Mutations in rhodopsin cause inherited retinal degenerative diseases such as retinitis pigmentosa. Several therapeutic strategies have attempted to address and counteract the deleterious effect of rhodopsin mutations on the conformation and function of this photoreceptor protein, but none has been successful in efficiently preventing retinal degeneration in humans. These approaches include, among others, the use of small molecules, known as pharmacological chaperones, that bind to the receptor stabilizing its proper folded conformation. Valproic acid, in its sodium valproate form, has been used as an anticonvulsant in epileptic patients and in the treatment of several psychiatric disorders. More recently, this compound has been tested as a potential therapeutic agent for the treatment of retinal degeneration associated with retinitis pigmentosa caused by rhodopsin mutations. We now report on the effect of sodium valproate on the conformational stability of heterologously expressed wild-type rhodopsin and a rhodopsin mutant, I307N, which has been shown to be an appropriate model for studying retinal degeneration in mice. We found no sign of enhanced stability for the dark inactive conformation of the I307N mutant. Furthermore, the photoactivated conformation of the mutant appears to be destabilized by sodium valproate as indicated by a faster decay of its active conformation. Therefore, our results support a destabilizing effect of sodium valproate on rhodopsin I307N mutant associated with retinal degeneration. These findings, at the molecular level, agree with recent clinical studies reporting negative effects of sodium valproate on the visual function of retinitis pigmentosa patients.

## 1. Introduction

Rhodopsin (Rho) is the photoreceptor protein found in the disk membranes of the rod outer segment (ROS) of retinal rod photoreceptor cells [1]. This protein is responsible for scotopic vision by converting photons into chemical signals triggering a biological response that enables the brain to sense light in the visual phototransduction process [2,3]. Rho is a key member of class A G protein-coupled receptors (GPCRs) and was the first GPCR whose crystallographic structure was solved at atomic resolution [4]. In fact, the Rho crystallographic structure has been used as a valuable model for structural studies of GPCRs [5,6], representing a structural basis for better understanding the structure-function relationships of other GPCRs [7,8]. GPCRs represent the largest family of membrane proteins, involved in most relevant physiological processes [9] and in mediating cellular responses elicited by extracellular signals for a proper cell function [10]. This receptor superfamily has been widely investigated due to the potential use of many of its members as pharmacological targets in drug discovery [11]. Mutations in GPCRs are associated with a number of pathologic conditions and are considered to be the cause of a wide range of human diseases [12]. In particular, mutations in the visual GPCR Rho are associated with inherited retinal degenerative diseases, particularly with retinitis pigmentosa (RP) [12].

RP is a class of known diseases including progressive degeneration of the rod and cone photoreceptor cells, usually starting in the mid-periphery and advancing toward the macula and fovea [13]. RP is a major cause of visual disability with a worldwide spread from 1:3000 to 1:7000 [13]. Generally, RP symptoms involve night blindness in adolescence followed by decreasing visual fields, leading to tunnel vision and eventually to legal blindness or, in some cases, to complete blindness [13]. Despite the majority of RP patients being non-syndromic, 20–30% of them have an associated non-ocular affectation [13]. The existence of a wide range of genes and mutations responsible for RP has hampered the development of targeted therapies. Several studies suggest that a dietary intake of diverse nutrients, such as vitamin A palmitate or docosahexaenoic acid, an ω-3 fatty acid, may slow down the disease progression in some forms of RP [14].

Other therapeutic strategies have focused on neuroprotection with several ongoing trials evaluating ciliary neurotrophic growth factor as a potential treatment for RP [15]. In the past years, valproic acid—in its sodium valproate form (VPA)—has been studied as a potential therapy for RP. Typically, VPA has been used as an anticonvulsant and mood stabilizer, and it is also known to cause gamma-aminobutyric acid (GABA) inhibitory effects in the central nervous system [16,17]. Moreover, VPA has also been pointed as a potential treatment for patients with retinal dystrophies due to its inhibitory effect on histone deacetylase [18], and on the inflammatory response pathway through apoptosis of microglial cells [19]. It has been hypothesized that VPA could act as a chaperone increasing the presence of correctly folded mutant Rho [20]. In addition, VPA is known to increase the levels of various neurotrophic factors and downregulate complement proteins [21,22]. However, despite these beneficial effects, VPA has been reported to have a number of undesired side effects, including hepatotoxicity and neurological and mitochondrial toxicity [23]. Therefore, the therapeutic benefits of VPA treatment on RP patients are still controversial and a definite answer is still lacking. Oral VPA administration to RP patients showed an improvement in visual field (VF) after an average of four months of treatment with acceptable adverse effects [24]. Other studies found that treatment with VPA had beneficial effects in other models of neurodegenerative diseases, possibly mediated by an effect on autophagy [25,26]. More recently, VPA has been associated with visual acuity (VA) and VF decline as well as adverse side effects in patients with pigmentary retinal dystrophies [17]. In a different study, an improvement in amplitude and latency/implicit time has been reported using multifocal electroretinography [27]. These controversial results underline the need for conducting further studies to get a deeper insight into the mechanism by which VPA would cause beneficial or detrimental effects in the treatment of RP.

Herein, we report the results of a comparative study designed to understand the effect of VPA on the conformational stability of native Rho from bovine retinas, recombinant wild-type (WT) Rho, and the I307N mutant associated with retinal degeneration in mice. Mutant I307N has been included in the study because it can be a good model for RP in humans and it may help to shed some light into the mechanism of the effect that VPA exerts on RP. To this aim, we have expressed WT and I307N mutant Rho, in eukaryotic cells, in the absence and in the presence of VPA. The expressed proteins have been regenerated with 11-cis-retinal (11CR) and purified by immunoaffinity chromatography in order to evaluate their conformational and stability properties. Native Rho, isolated from bovine retinas, was used as a control sample for our experiments.

Only moderate effects of VPA, either on the dark thermal stability or on the photoactivated receptor decay rates, on native ROS and WT Rho proteins were observed. However, the I307N mutant showed faster thermal decay rates than the WT in the untreated samples due to a lower conformational stability of the RP mutant. In addition, the VPA-treated mutant receptor showed a faster decay process after illumination, which reflected a significant decrease in its active conformation stability. In conclusion, our results indicate a destabilizing effect of VPA on the Rho retinal degeneration mutant that supports recent clinical studies reporting negative effects of the compound on the visual function of RP patients [28].

## 2. Results

### 2.1. UV-Vis Spectroscopic Characterization.

The results of ROS Rho, WT, and mutants with and without VPA, were analyzed by means of UV-Vis spectrophotometry as described under Materials and Methods. A summary of the spectral parameters, including the absorbance value of the visible chromophoric band, ε, and the spectral A_280_/A_λmax_ ratio, is shown in Table 1. A higher A_280_/A_λmax_ can be detected for the VPA containing samples in all cases.

**Figure 1:**
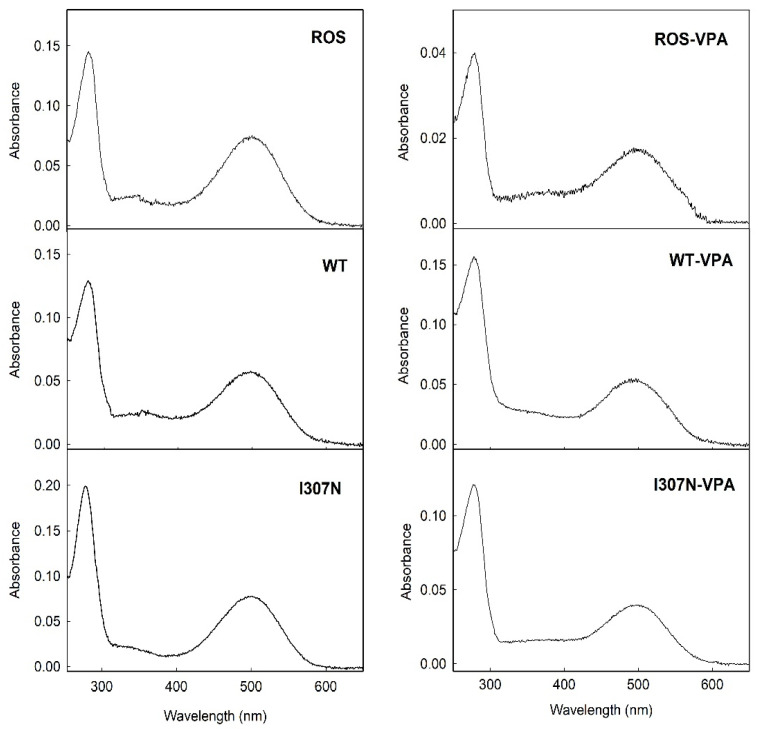
UV-vis absorption spectra of immunopurified ROS, WT, and I307N mutant Rhos, with and without VPA treatment, in the dark state. Samples are in buffer B (137 mM NaCl, 2.7 mM KCl, 1.5 mM KH_2_PO_4_, and 8 Mm Na_2_HPO_4_, pH 7.4, 0.05% DM). Spectra were recorded at 20 °C with the spectral parameters described under the Materials and Methods section.

WT Rho and the I307N mutant were expressed and immunopurified, and the UV-vis spectra of the purified samples were recorded at 20 °C. The UV-vis spectra of native ROS Rho and those of the WT and the I307N mutant, show similar features with and without VPA treatment (Figure 1). The WT and the I307N mutant without VPA showed a spectroscopic behavior similar to ROS Rho, with a maximum absorption band in the visible region at 500 nm in agreement with previously reported spectra [29]. Samples treated with VPA showed a small blue shift of 3 nm in their visible band with respect to those samples without VPA treatment. WT Rho showed similar spectroscopic parameters, without and with VPA treatment, to those of ROS Rho with Amax of 500 and 497 nm, respectively (Table 1). The A_280_/A_λmax_ ratio of WT Rho was increased from 2.22 ± 0.20 to 2.89 ± 0.20 in the VPA treated samples. A similar increase was detected for the ROS Rho sample with A_280_/A_λmax_ ratios of 1.90 ± 0.12 and 2.83 ± 0.24, respectively. In the case of the I307N mutant, the A_280_/A_λmax_ ratio was 2.55 ± 0.30 in the non-treated sample and 3.02 ± 0.3 in the VPA-treated sample. The molar extinction coefficients of ROS, WT, and I307N mutants were similar in the three cases but were slightly reduced in the VPA-treated samples.

### 2.2. Photobleaching and Acidification

**Figure 2:**
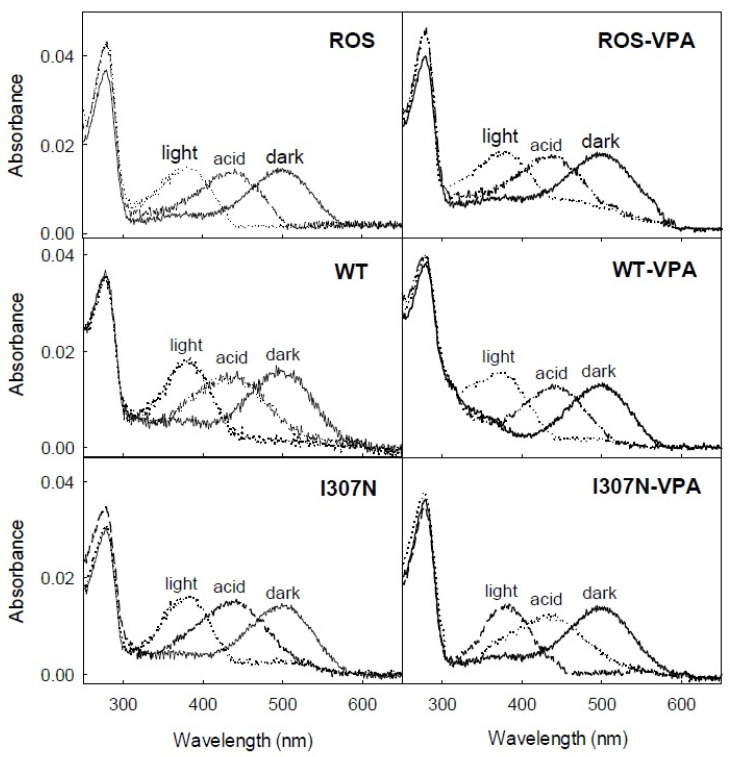
UV-vis photobleaching and acidification of the immunopurified ROS, WT, and RP mutant. Dark state (solid line), photobleached (dashed line), and acidified (dotted line). Samples in buffer B (137 mM NaCl, 2.7 mM KCl, 1.5 mM KH_2_PO_4_, and 8 Mm Na_2_HPO_4_, pH 7.4, 0.05% DM). Spectra were recorded at 20 °C.

Photobleaching of Rho can be followed by the blue-shift of the 500-nm chromophoric band in the visible region to 380 nm. This shift is due to the SB nitrogen deprotonation in the Meta II state. The UV-vis spectra of ROS and WT Rho purification expressed in the presence and in the absence of VPA were recorded in the dark, upon illumination for 30 s and after subsequent acidification (Figure 2).

The main difference observed was in the I307 mutant with VPA that did not show a complete conversion of the visible band to 380 nm upon illumination. The presence of the remaining absorbance in the visible region (about 30% of the dark visible band) suggests partial conversion to a photointermediate with a protonated SB (PSB) linkage [30]. No significant differences could be observed in the spectral features of the ROS Rho and WT Rho samples treated with VPA upon illumination. Acidification of ROS and WT with and without VPA treatment resulted in a similar behavior, showing a band with an absorbance maximum at 440 nm corresponding to acid trapping of the PSB.

### 2.3. Thermal Stability

**Figure 3:**
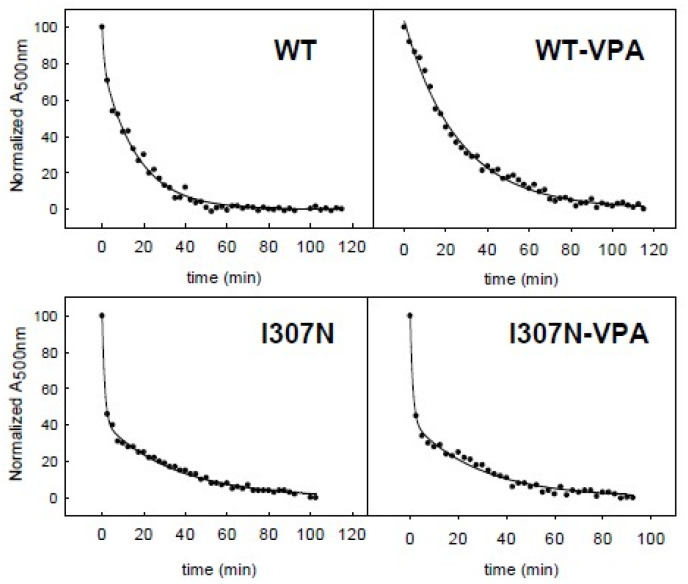
Representative curves for WT and I307N mutant thermal stabilities, in the dark at 48 °C, with and without VPA treatment. The experimental absorbance values at 500 nm were normalized and curve-fitted to an exponential function with Sigma Plot 12.5.

**Figure 4:**
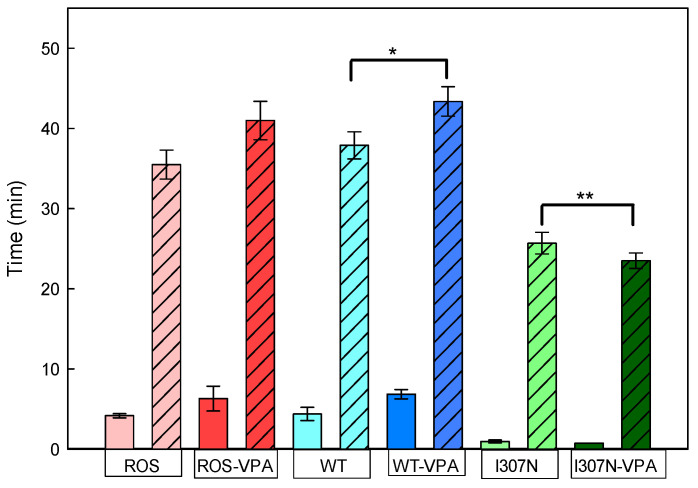
Thermal stability of the immunopurified ROS, WT, and I307N mutant Rhos at 48 °C. The thermal decay of the visible band was followed with time and the corresponding curves were fit to an exponential decay function with two components. The t_1/2_ for the fast (solid color) and the t_1/2_ for the slow component (hatched bar) were derived and are plotted for the non-treated (ROS—pink; WT—light blue; I307N—light green) and VPA-treated samples (ROS—red color; WT—dark blue; I307N—dark green) as indicated in the corresponding labels. The mean value and standard error (SE) were obtained in independent purifications (*n* = 3). Treatment with VPA results in a small, but significant, increase in the thermal stability of WT Rho (*), and a small but non-significant decrease in the case of the I307N Rho mutant (**) (*p* < 0.05).

The thermal stability of ROS, WT, and I307N mutant samples with and without VPA treatment was followed by measuring the decrease in the Amax in the visible region over time at the constant temperature of 48 °C (Figure 3 and Figure 4; Table 2). ROS Rho could be bleached in the dark as a result of increasing the temperature and this would force chromophore isomerization. Such thermally-induced retinal isomerization consists of two steps. First, thermal isomerization of 11CR in the binding site of Rho yields all-trans-retinal (ATR) bound to opsin, followed by hydrolysis of the deprotonated SB yielding free ATR and opsin [31] at a second stage. The thermal decay curves were best fit to double exponential functions (Figure 3) which gave two t_1/2_ decay times corresponding to a fast and a slow component, respectively (T1 and T2). Thus, the thermal decay times for ROS Rho, with and without VPA, showed t_1/2_ (T1) similar in the case of the non-treated and VPA-treated samples (6.3 ± 1.5 and 4.2 ± 0.3 min, respectively). These times were similar to those of recombinant WT Rho (6.8 ± 0.6 and 4.4 ± 0.6 min). However, the I307N mutant, both with and without VPA treatment, showed a very unstable conformation in the dark state as seen from fast thermal bleaching kinetics with 0.9 ± 0.2 and 0.7 ± 0.1 min, respectively, for the first t_1/2_ (T1) (Figure 4). Furthermore, the I307N mutant showed a t_1/2_ (T2) that was nearly two-times faster than that of WT Rho both in the untreated and treated samples (Table 2). These results indicate a reduced thermal stability of the I307N mutant when compared either to ROS or to WT Rho for both the T1 and T2 half-life times in the non-treated samples. VPA treatment slightly reduced the thermal stability of the I307N mutant compared to ROS and WT Rho cases, but the result was not statistically significant (Table 2).

### 2.4. Meta II Decay

**Figure 5:**
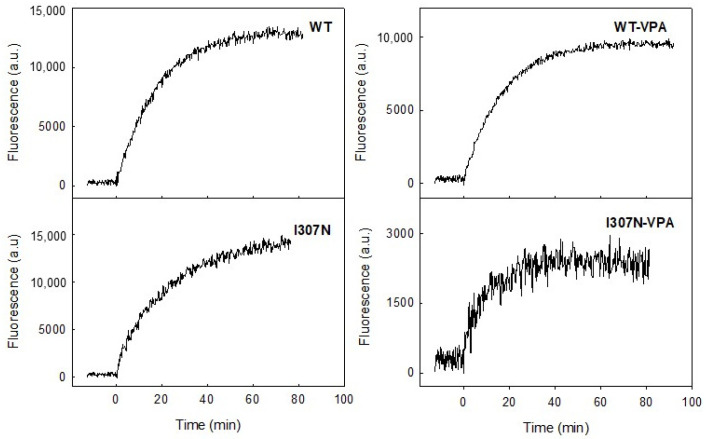
Fluorescence Retinal Release Curves: The figure shows representative curves for the purified Rho WT and mutant samples measured by means of fluorescence spectroscopy (the I307N mutant without and with VPA treatment is shown in comparison to WT Rho). The fluorescence curves represent the retinal release from the Rho binding pocket which closely parallels the Meta II decay process. The protein samples were incubated at 20 °C until a steady baseline was obtained, they were subsequently photobleached and the Trp fluorescence increase was monitored over time. The fluorescence increase curve was fit to a single exponential function and the t_1/2_ obtained.

**Figure 6:**
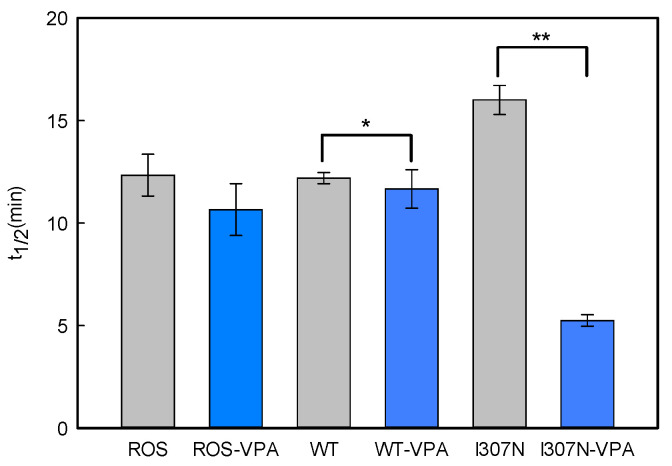
Fluorescence Meta II Decay: The t_1/2_ for the retinal decrease that closely parallels de Meta II decay process under our experimental conditions was determined from experimental results depicted in Figure 4. The t_1/2_ values for immunopurified ROS, WT, and I307N mutant, with (dark blue) and without VPA treatment (grey), are reported. The mean value and standard error (SE) were obtained from independent purifications (*n* = 3). Treatment with VPA does not cause a significant effect on the active conformation stability of WT Rho (*) but a statistically significant decrease in the stability of the Meta II conformation of the I307N mutant (**) (*p* < 0.05).

The stability of the active conformation of purified ROS, WT, and I307N mutant Rhos was determined by means of steady-state fluorescence spectroscopy. In the dark state, Trp265 fluorescence is quenched by the β-ionone ring of the retinal and, upon illumination, retinal is released from the protein binding pocket thereby resulting in an increase in the intrinsic Trp265 fluorescence emission. This retinal release process (which closely parallels the Meta II active conformation decay under our experimental conditions) can be followed at 330 nm for an excitation wavelength of 295 nm. The fluorescence changes were monitored continuously over time after photobleaching. To determine the t_1/2_ values for retinal release, experimental fluorescence data was analyzed using a mono-exponential rise to maxima fit (Figure 5 and Figure 6) and the corresponding t_1/2_ values were derived from each curve.

The Meta II t_1/2_ values, obtained from the fluorescence curves, were very similar for the ROS and WT but showed some increase for the mutant in the untreated samples (Figure 6). A previous study showed a similarly increased but slightly longer t_1/2_ for the I307N mutant [32]. This result indicates that the I307N mutation affects the active conformation of the protein and that this conformational rearrangement appears to enhance the life-time of the active conformation rather than destabilizing it. However, the I307N mutant showed a clearly faster decay process suggesting a de-stabilized active Meta II conformation as a result of VPA treatment (from 16.3 ± 0.6 to 5.2 ± 0.2 min).

### 2.5. Molecular Modelling Analysis

**Figure 7:**
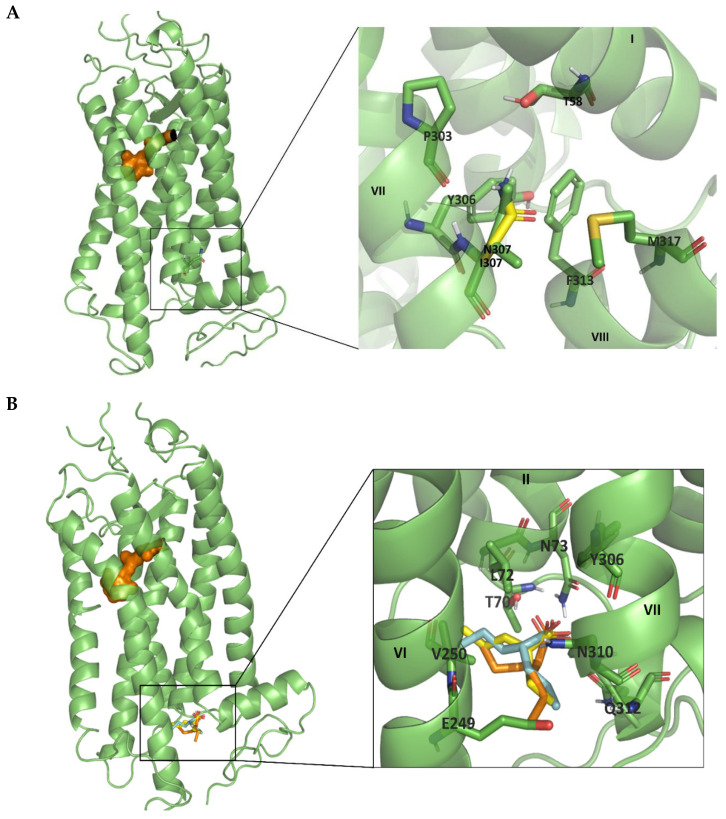
(**A**)**.** Structure of Rho receptor, PDB code 1U19, focusing on the position of Ile307. Detailed representation of the Ile307 position and the nearby residues can be observed on the right, where the mutation to Asn is represented in bright yellow. (**B**). Potential VPA binding site to the intracellular domain of Rho. Positions of the best three poses of VPA docking, with values of the scoring function of −4.5, −4.4, and −4.4 kcal/mol, respectively, are represented in cyan, bright yellow, and orange.

Computational studies were carried out to identify prospective binding sites of VPA to Rho and the I307N mutant. First, we analyzed the structural effects caused by the mutation of Ile to Asn in the I307N mutant. In WT Rho, Ile307 may be stabilizing the inactive conformation of the receptor by means of hydrophobic interactions with Tyr306 and Phe313. In the I307N mutant, analysis of the structure reveals residue Asn307 exhibiting hydrogen bonding interactions with nearby residues Thr58 and Met317 that are not possible for Ile307 in the WT. In both cases, Asn307 acts as a hydrogen donor with the other two residues as can be seen in Figure 7A. Subsequently, we analyzed the results of the docking analysis to identify prospective binding sites for VPA at the intracellular site of the receptors. Once the different binding sites of VPA were rank ordered, the preferred site is located between helices TM2, TM6, and TM7 (Figure 7B). The binding site is close to Asn307 and this could explain the effect of the mutation on the stability of the I307N mutant. Specifically, it can be hypothesized that VPA may affect the flexibility of TM7 and consequently affect the interactions of Asn307 with residues Thr58 or Met317, somehow facilitating the transition to the active conformation of the receptor. In any event, a detailed molecular dynamics study should help clarify the structural features of VPA binding to Rho and the effect on the mutant receptor.

## 3. Discussion

Most of the studies regarding effects of Rho mutations associated with RP have been performed in the background of bovine Rho [27]. However, small amino acid sequence differences in Rho have been shown to have physiological relevance [33]. We have observed different protein phenotypes for Rho mutants depending on the species considered (human, bovine, and murine). Recently, differential aggregation properties have been reported for Rho mutants expressed in human and bovine genes [34]. Therefore, it is increasingly evident that human Rho is a more reliable model for the study of pathogenic mutations. In spite of this, many studies dealing with RP mutations have been conducted mainly using transgenic mice [35] but using transgenes can complicate the analysis of the results due to overexpression of the WT allele. In a particular case, mutagenized mice with Y102H and I307N Rho mutations have been shown to suffer retinal degeneration [32] and to be valuable models that may help avoid the problems associated with studies using transgenic models [29]. Specifically, the I307N mutant has been shown to be valuable in preclinical and mechanistic studies [36], in spite of the potential differential effects in the human background. Bearing this in mind, we decided to use this mutant to explore the effect of VPA on its biochemical and biophysical properties. To this aim, we first characterized the spectroscopic properties of the mutant without VPA treatment and compared them with those of ROS Rho and WT Rho expressed in mammalian cells. Our results showed a higher A_280_/A_λmax_ ratio for the I307N mutant in agreement with previously reported data [32], which suggest that this mutation can cause the production of misfolded protein and/or a decrease in the structural stability of the mutated protein.

Our spectroscopic analysis shows that the properties of dark state conformations of I307N mutant, ROS, and WT Rho were only slightly affected by VPA, and only a higher A_280_/A_λmax_ ratio was detected in the treated samples. This might be due to some fraction of misfolded protein and/or protein with decreased structural stability that can be slightly increased upon VPA treatment. Changes in this ratio may also reflect some minor aggregation in the treated samples. A small but detectable blue-shift could be detected for the WT and I307N mutant in the visible band in the treated samples. Photobleaching and acidification results suggest no important differences in the photochemical properties of Rho. In agreement with these results, a slight shift like the one in the treated sample has been detected in studies with other Rho mutants and attributed to minor structural perturbations of the protein [33,37].

In terms of the dark-state conformational stability, we found a great reduction in the thermal stability of the I307N mutant with respect to ROS and WT Rhos suggesting a lower conformational stability of the mutant, in agreement with previous results [32,38]. Furthermore, there was no sign of increased stability from the dark inactive conformation of the mutant upon treatment with VPA. The mutant is intrinsically less stable than WT Rho in the dark state and this is also detected in the presence of VPA, showing a faster thermal bleaching process [32]. In contrast, ROS and WT samples showed a small increase in the stability of the protein upon VPA treatment (Table 2). The observed behavior can be rationalized considering that Rho can be activated in the dark by increasing the temperature which would force chromophore isomerization [39,40]. Our results show a small increase in the thermal stability of the receptor decay rates for ROS and WT proteins upon VPA treatment, but an apparent, although not significant, decrease in the case of the mutant (Figure 4). The mutant behavior could be related to a structural change that may have an impact on the adjacent NPXXY structural motif and/or on helix 8 and the C-terminal tail of Rho whose contribution to the stability of the correctly folded conformation of the protein has been usually underestimated. The I307N mutant also shows interesting features in contrast to the physicochemical properties of the WT photoreceptor protein. In this regard, the high percentage and rate of chromophore regeneration together with the slower retinal release in this mutant [38] could be related with the uptake and release of the retinal through a proposed retinal channel. According to the Meta II crystal structure, the retinal must go through a complex elongation and torsional motions of its polyene chain and of the β–ionone ring during its binding process [41].

Regarding the functional conformation of the I307N mutant, VPA appears to accelerate the decay of its active Meta II conformation, obtained upon illumination, maybe due to an alteration of the active–inactive conformational equilibrium of the protein. Our molecular modeling analysis also suggests that the new interactions present in the I307N Rho mutant might be affecting active conformational state of the receptor, but the physiological correlation of this assumption is missing, and further simulations studying the activation of these mutated models must be performed to better define the mutational effect. How this effect could be correlated with in vivo results previously reported would require further investigation.

A relevant question arises regarding the molecular mechanism of VPA action in visual phototransduction. It should be noted that a proposed mode of action of VPA would involve blockade to ion channels but our study was aimed at investigating the potential direct effect on the visual photoreceptor Rho. This is part of an ongoing effort to find novel ligands (or find novel effects of known molecules) that could be used to off-set the deleterious effect of pathogenic mutations in Rho [41]. The fact that the I307N has been shown to have basal activity in the dark [38] makes the study of downstream signaling alterations an interesting new avenue of research worth pursuing in a subsequent more detailed study.

From our analysis, we have determined that VPA does not increase the conformational stability of either the inactive or the active conformations of I307N Rho mutant associated with retinal degeneration. In vivo studies have reported contradicting results. Vent-Schmid et al. reported that treatment with VPA reduced the abundance of mutant Rho in rod photoreceptors. Interestingly, VPA treatment did not provide complete rescue of inherited retinal degeneration either alone or in combination with dark rearing [42]. A more recent clinical study showed negative effects on RP patients treated with VPA and worse visual function than those of the placebo group [25]. This finding emphasizes the need to carefully consider key methodological considerations that must be applied to the intensive evaluation of treatments for these retinal degenerative conditions. This study clearly did not provide support for the use of VPA in the treatment of autosomal dominant RP patients [28]. Another clinical study evaluating the efficacy and safety of VPA treatment in patients with RP, on a total of 48 eyes of 24 patients, showed no significant benefit on the best-corrected visual acuity and the visual field analyses [17]. In addition, the VPA effect might have been preliminary associated with decline in some multifocal electroretinography parameters. Therefore, this study recommended that physicians should avoid prescribing VPA for RP until its safety and efficacy were appropriately evaluated [17]. In another study, no effect on retinal function was observed and no periodic retinal assessment was recommended in patients taking this medication [43].

## 4. Materials and Methods

### 4.1. Materials

All chemicals were reagent grade and were purchased from either Fisher (Darmstadt, Germany) or Sigma (Madrid, Spain). 11CR was provided by the National Eye Institute, National Institutes of Health (Bethesda, MD, USA), mAb Rho-1D4 antibody was obtained from Cell Essentials (Boston, MA, USA) and was coupled to cyanogen bromide (CNBr)-activated Sheparose 4B beads from Sigma (Spain). *n*-Dodecyl-β-d-maltoside (DM) was from Anatrace Inc. (Maumee, OH, USA), H-TETSQVAPA-OH (9-mer) peptide was synthesized by Unitat de Tècniques Separatives i Síntesi de Pèptids (Barcelona, Spain), polyethyleneimine (PEI) was purchased from Polysciences Inc. (Warrington, PA, USA). Dulbecco’s modified Eagle medium (DMEM) supplemented with fetal bovine serum, L-glutamine, and penicillin-streptomycin was applied to culture COS-1 cells. COS-1 cells were obtained from the American Type Culture Collection (Manassas, VA, USA). VPA was purchased from Sigma (Madrid, Spain). The following solutions were used: buffer A (137 mM NaCl, 2.7 mM KCl, 1.5 mM KH_2_PO_4_, and 8 Mm Na_2_HPO_4_, pH 7.4); buffer B (buffer A with 0.05% DM).

### 4.2. Methods

#### 4.2.1. ROS Rho Purification

ROS membranes were prepared, from frozen bovine retinas, under dim red light using a sucrose-gradient method as described previously [44]. The isolated ROS membranes were suspended in KH_2_PO_4_ (70 mM, pH 6.9), MgCl_2_ (1 mM), and EDTA (0.1 mM), and were centrifuged for 30 min at 35,000 rpm in a 45Ti rotor. The membrane pellets were resuspended in Tris-HCl (5 mM, pH 7.5) containing MgCl_2_ (0.5 mM). The pellet was washed twice with this buffer to minimize the presence of contaminating proteins. The obtained product was split into several aliquots and stored in the dark at –80 °C. For purification, the ROS membranes were previously solubilized by incubating in buffer A containing 1% DM in the dark for 1 h at 4 °C, and the solution was subsequently subject to centrifugation (3800 rpm, 40 min). The supernatant was collected and Rho was purified by immunoaffinity chromatography on CNBr-activated sepharose resin coupled to Rho-1D4 antibody. The purified protein was eluted in buffer B containing 100 μM of 9-mer peptide [45,46].

#### 4.2.2. Construction of Opsin Mutant

The I307N mutant was designed by means of site-directed mutagenesis (QuikChange, Stratagene) on a synthetic bovine opsin gene [47] using the pMT4 vector as a template. The following primers were used for the I307N mutation (ATC → AAC):

forward primer: CCCGGTCATCTACAACATGATGAACAAGCAGTTCC;
reverse primer: GGAACTGCTTGTTCATCATGTTGTAGATGACCGGG.

Primers were obtained from Sigma (Spain). The mutant plasmid was sequenced to verify the correct introduction of the mutation.

#### 4.2.3. Expression and Purification Bovine Recombinant WT Rho and I307N Mutant Genes

The experimental procedures for the expression and purification of recombinant Rho have been previously described [38]. Briefly, the WT opsin and I307N mutant genes, in the pMT4 plasmid, were expressed in eukaryotic COS-1 cells at 85% confluence with 30 μg of plasmid DNA per 15-cm plate. PEI reagent (100 μL at 1 mg/mL) was used to transfect genes into the eukaryotic cells. For VPA treatment, VPA was added to the cell culture medium, from a stock solution in buffer A, to a final concentration of 3 μM. After 48 h, the cells were harvested and the medium was removed. To completely remove the culture medium and VPA, the cells were washed twice with 15 mL of buffer A. Opsins were subsequently regenerated with 15 μM 11CR in buffer A by overnight incubation at 4 °C. Then cells were solubilized using 1% DM with 100 μM phenylmethylsulphonyl fluoride (PMSF), and protease inhibitor cocktail, by shaking for 1 h at 4 °C, followed by ultracentrifugation for 30 min at 35,000 rpm. WT and I307N mutant were immunopurified from the supernatant using CNBr-activated sepharose resin coupled to Rho-1D4 antibody. The resin was washed with buffer B and the bound pigments were subsequently eluted with buffer B containing 100 μM 9-mer peptide. All processes were carried out in the dark or under dim-red light and the samples were always kept on ice. The concentration of the purified Rho samples was determined by means of their UV-vis spectra and the corresponding protein concentrations were in the 0.5–1.5 μM range depending on the assay conducted. Therefore, the Rho/VPA molar ratio ranged from 1:2 to 1:6 depending on the type of assay conducted. The use of different VPA concentrations would be required for getting a deeper insight into the VPA effects reported in the current study.

#### 4.2.4. UV-Visible Spectral Characterization

For spectroscopic characterization of the Rho samples, a Varian Cary 100 Bio spectrophotometer (Varian, Australia) was used. Temperature was controlled by means of a peltier accessory equipped with a water-jacketed cuvette holder connected to a circulating water bath. All spectra were registered in the 250–650 nm range with a bandwidth of 2 nm, a response time of 0.5 s, and a scan speed of 400 nm/min.

#### 4.2.5. Photobleaching and Acidification of Purified Rho.

Samples were illuminated with a 150-W Dolan-Jenner MI-150 power source, equipped with an optic fiber guide and using a 495-nm cut-off filter, for 90 s to ensure complete photoconversion of the visible chromophoric band to the 380-nm absorbing species. Acidification was performed, immediately after photobleaching, by the addition of 2 N H_2_SO_4_ which yields a pH ~2.0 and the absorption spectrum was subsequently recorded. The reprotonated Schiff base (SB) caused by acidification shifts the Amax of the visible band to 440 nm.

#### 4.2.6. Thermal Stability

Thermal stability of Rho was examined by monitoring the decrease of Amax of the visible spectral band as a function of time at 48 °C. Spectra were registered every 5 min and half-life times were derived by fitting the experimental data to exponential curves.

#### 4.2.7. Metarhodopsin II (Meta II) Decay

Meta II decay rates were determined from fluorometric measurements on a QuantaMaster 4 spectrofluorometer (Proton Technology International, Edison, NJ, USA) equipped with a TLC50 cuvette holder peltier accessory, for temperature control. Firstly, Trp fluorescence of a dark-adapted sample was recorded at 20 °C until a stable base line was obtained. Then, the sample was illuminated for 30 s with a 150-W Dolan-Jenner MI-150 power source using a cut-off filter (>495 nm) and the fluorescence intensity was monitored until it reached a plateau. All fluorescence spectra were performed by measuring the fluorescence signal of the samples for 2 s at 295 nm, with a slit bandwidth of 0.5 nm, and blocking the excitation beam for 28 s with a beam shutter to avoid photobleaching of the sample. Trp emission was monitored at 330 nm with a slit bandwidth of 10 nm. The fluorescence curve was fit to an exponential function and the half-life time (t_1/2_) values of the decay process were derived.

#### 4.2.8. Molecular Modelling Studies

The three-dimensional structure of Rho was retrieved from the Protein Data Bank (PDB) (PDB ID: 1U19). This structure was also used to construct the 3D structure of the I307N mutant using PyMOL [48]. The procedure permits the construction of a few models of the mutant Rho with the side chain of the substituted residue in different conformations selected from a library of rotamers. Hydrogens were added to the WT and mutant structures using the LEaP module of the AMBER18 software [49]. Energy minimization of the systems was carried out using the steepest descent (SD) algorithm [50]. Docking studies of VPA onto the Rho receptor and mutant were carried out with Auto Dock [51]. Specifically, the structures of either the Rho receptor or the I307N mutant were imported, after energy minimization, into the Auto Dock Tools interactive graphical tool. Subsequently, a box with appropriate dimensions was built at the intracellular side of the receptor. Then, docking of different conformations of VPA was performed using QuickVina2 (QVina2), from the Auto Dock Vina program [52,53], which allows one to classify and assign the scoring function of different molecules, or in this particular case conformations, in a fast way, obtaining the best one in energy terms.

#### 4.2.9. Data Analysis

Results were reported as the mean value ± standard error (SE) obtained from independent purifications (*n* = 3) and analyzed using Sigma Plot 12.5 (Systat Software Inc. San Jose, CA, USA). Statistical significance was determined by unpaired two-tailed Student’s *t*-test using *p*-value < 0.05.

## 5. Conclusions

The results presented on the biochemical and biophysical properties of the purified I307N mutant protein do not indicate any improvement in the conformational stability of the protein. Neither the inactive dark state conformation nor the active conformation of this mutant Rho, were found to be stabilized by treatment with VPA. On the contrary, a clear fastening of the Meta II decay process, which could be interpreted as a destabilized active conformation, was clearly observed in the case of the I307N mutant. Our results would agree with the clinical findings that reported negative effects of VPA on visual function of RP patients [25] and provide a structural correlation that deserves further investigation in order to clarify the link between the photoreceptor protein function and the physiological effects observed. The use of more specific cell lines should be helpful in obtaining novel information on the investigated effects of pathogenic Rho mutations. Our results open up new interesting questions such as the effect of VPA on downstream signaling elements of Rho. This aspect, and a thorough analysis of the protein features for different protein and VPA concentrations, would require further investigation.

## Figures and Tables

**Table 1:** UV-Vis Spectroscopy: UV-vis spectral parameters of ROS, WT, and I307N mutant Rhos, with and without VPA treatment, in the dark state.

	ROS	ROS-VPA	WT	WT–VPA	I307N	I307N-VPA
λ_max_ (nm)	500	501	500	497	500	497
A_280_/A_λmax_	1.90 ± 0.10	2.83 ± 0.20	2.22 ± 0.20	2.89 ± 0.20	2.55 ± 0.30	3.02 ± 0.30
ε × 10^3^ (M^−1^·cm^−1^)	42.7 ± 1.1	37.6 ± 1.8	43.2 ± 0.6	37.5 ± 2.2	43.8 ± 0.3	37.8 ± 2.5

**Table 2:** Thermal Decay Rates: Results of the first (T1) and second (T2) half-life times (t_1/2_) for the thermal stability of the purified ROS, WT, and I307 mutant Rhos, in the dark state. The decrease of the visible absorbance maximum with time was followed at 48 °C in the dark and the half-life times for the thermal decay were determined as described under the Materials and Methods section.

	ROS	ROS-VPA	WT	WT-VPA	I307N	I307N-VPA
T1 (min)	4.2 ± 0.3	6.3 ± 1.5	4.4 ± 0.6	6.8 ± 0.6	0.9 ± 0.2	0.7 ± 0.1
T2 (min)	35.5 ± 1.8	41.1 ± 2.4	38.1 ± 1.7	43.4 ± 1.8	25.7 ± 1.3	23.5 ± 0.1

## Data Availability

The data presented in this study are available on request from the corresponding author.

## Sample Availability

Samples of the plasmid harboring the I307N mutation in bovine Rho are available from the authors.
